# A newly identified photosystem II Subunit P gene *TaPsbP4A-1* in Triticeae species negatively regulates wheat powdery mildew resistance

**DOI:** 10.3389/fpls.2024.1452281

**Published:** 2024-11-08

**Authors:** Jun Xu, Mengfei Wang, Yueming Ren, Wanglong Luo, Lu Zhang, Shuangwei Liu, Ping Hu

**Affiliations:** ^1^ College of Horticulture and Landscape Architecture, Henan Institute of Science and Technology, Xinxiang, China; ^2^ College of Agriculture, Henan Engineering Research Center of Crop Genome Editing/Henan International Joint Laboratory of Plant Genetic Improvement and Soil Remediation, Henan Institute of Science and Technology, Xinxiang, China

**Keywords:** photosystem II Subunit P, wheat powdery mildew, expression pattern, barley stripe mosaic virus-induced gene silencing, evolutionary progress

## Abstract

The photosystem II (PSII) Subunit P (PsbP) protein is a component of its oxygen-evolving complex, which can oxidize water to produce oxygen using light energy and is critical to the core components and stability of PSII. Using the whole-genome information, the *PsbP* genes of 10 plant species were comprehensively identified. The expression patterns of wheat *PsbP*s under *Blumeria graminis* f. sp. *tritici* (*Bgt*) infection were assessed using qRT-PCR, and the functions of *TaPsbP*s in wheat powdery mildew resistance were studied using barley stripe mosaic virus-induced gene silencing. In total, 122 *PsbP* genes were divided into 8 classes with similar gene structures. No tandem repeat events were identified in wheat *PsbP*, suggesting that the *PsbP* genes in common wheat were donated by its diploid progenitor species. The expression levels of *TaPsbP2A-1*, *TaPsbP3A-1*, *TaPsbP4A-1*, *TaPsbP4A-2*, and *TaPsbP7A-2* were induced by *Bgt*. The silencing of *TaPsbP4A-1* increased the resistance of common wheat ‘Bainong AK58’ to *Bgt*. This study provides valuable information for functional and evolutionary research on the *PsbP* gene family.

## 1 Introduction

Plants and algae possess the photosystem II (PSII) complex, which uses light energy to oxidize water into molecular oxygen (Ifuku et al., 2008). The PSII Subunit P (PsbP: OEC23) protein is a component of the oxygen-evolving complex (OEC) of PSII, and the other two nuclear-encoded proteins of OEC, PsbO (OEC33), and PsbQ (OEC16), constitute an indispensable external protein domain in green algae and higher plants PSII (Miyao and Murata, 1985; Gnanasekaran et al., 2019; Hong et al., 2020). PsbP and PsbQ in higher plants originated from cyanobacterial cyanoP and cyanoQ, respectively (De Las Rivas and Roman, 2005; Ishihara et al., 2007). The lipid structures at their N-termini help them anchor to the thylakoid membrane, thereby helping calcium and chloride ions bind to the PSII complex. The high-resolution structures of Tobacco PsbP (1.6A˚) and Spinach PsbP (1.98A˚) revealed that the core structures are antiparallel β-sheets with both sides having α screws (Ifuku et al., 2004; Kopecky et al., 2012). PsbP family proteins can be divided into eight subclasses (Class a –h). Along with the PsbP proteins, two PsbP-like (PPL1 and PPL2) proteins and seven PsbP-domain (PPD) proteins, constitute the *Arabidopsis* PsbP protein family (Ifuku et al., 2008; Sato, 2010; Bricker et al., 2013).

PsbP family proteins in plants have different functions. After PsbP is synthesized on the cytoplasmic ribosomes, it is transported to the cystoid cavity in the chloroplast by a transport peptide (Bricker et al., 2013). PsbP regulates the water decomposition reaction, which is crucial to PSII core assembly and stability. Knockdowns of *PsbP* in *Arabidopsis* and tobacco revealed that PsbPs are indispensable to the stabilization of PSII (Yi et al., 2009). The dissociation of the PsbP protein leads to a decrease in PSII activity, and a decrease in its expression level leads to a decrease in photosynthesis (Ido et al., 2009; Kong et al., 2014; Liu et al., 2021). *Arabidopsis PPL1* effectively repairs photo-damaged PSII under high-intensity light, *AtPPL2* plays an important role in NAD(P)H dehydrogenase complex accumulation (Ishihara et al., 2007), and *AtPPD1* plays a role in assisting PSI assembly (Liu et al., 2013; Hong et al., 2020).

The salt-stress treatment of PSII reaction centers in spinach *in vitro* leads to the complete dissociation of the PsbP protein and a reduction in the PSII activity (Nishimura et al., 2017). An SDS-PAGE analysis showed that the PsbP protein on a leaf thylakoid membrane decreases significantly after 45 days of 400-mM NaCl treatment (D’Andrea, 2003). Under salt-stress conditions, compared with the salt-sensitive soybean ‘Jackson’ variety, the salt-tolerant ‘Lee 6’ variety has a higher PsbP protein content, which contributes to the stability of the OEC structure (Carvalho et al., 2006). The chloroplast protein PPD5 regulates the accumulation of H_2_O_2_ in protective cells through the open stomata 1-dependent pathway and negatively regulates the drought resistance of *Arabidopsis* (Hong et al., 2020). Additionally, the accumulation of PsbP protein increases in *Ipomoea batatas* under heat stress and is stabilized by Orange protein to protect it from heat-induced denaturation (Kang et al., 2017).

For biotic stresses, after infection by *tobacco mosaic virus*, the PsbP accumulation in tobacco leaves significantly decreases (Rahoutei et al., 2000; Pérez-Bueno et al., 2004). In addition, *PsbP* overexpression reduces the accumulation of alfalfa mosaic virus, whereas *PsbP* silencing increases the severity of rice stripe virus infection and stimulates the accumulation of gemini viruses in tobacco (Balasubramaniam et al., 2014; Kong et al., 2014; Gnanasekaran et al., 2019). The effector RXLR31154 of grape downy mildew fungus stabilizes PsbP, reduces H_2_O_2_ accumulation, activates the ^1^O2 signaling pathway, and promotes the occurrence of plant host diseases (Liu et al., 2021). After being infected with downy mildew, the PsbP protein accumulation in grapes undergoes significant changes (Milli et al., 2012; Nascimento-Gavioli et al., 2017). Although there are reports on the functions of PsbPs, their evolution and molecular mechanisms are still unclear.

Bread wheat (*Triticum aestivum* L.), as an important source of Carbohydrate source, is subjected to many kinds of stress during its growth, among which powdery mildew, as a serious disease of wheat, poses a great threat to wheat yield and quality (Hu et al., 2018; Xing et al., 2018; Schilling et al., 2020). With the drastic changes in the global climate, the incidence area of wheat powdery mildew is expanding, and the use of traditional chemical control methods has caused great environmental pressure. Using molecular biological methods to explore the disease-resistance genes and develop disease-resistant varieties are environmentally friendly and effective goals of plant breeding (Bai and Shaner, 2004; Kuraparthy et al., 2007; Dean et al., 2012; Hu et al., 2023). At present, there has been limited research on the roles of *PsbP* in response to biotic stresses. Therefore, we used bioinformatics methods to identify the *PsbP* genes in six monocotyledon and four dicotyledon at the whole-genome level and analyzed the gene structures, evolution, duplication and expression patterns. Furthermore, the functions of three *TaPsbP*s in wheat powdery mildew were analyzed using barley stripe mosaic virus-induced gene silencing (BSMV-VIGS). These results provide a reference for the application of *PsbP* genes in wheat powdery mildew disease-resistance breeding.

## 2 Materials and methods

### 2.1 Plant treatment

The experiments were conducted in Xinxiang, Henan province, China, where *Blumeria graminis* f. sp. *tritici* (*Bgt*) mixed race was collected from the field and stored on seedlings of susceptible variety ‘Sumai 3’. The *Bgt* was stored in a climate chamber with 70% relative humidity for 14-h light/22°C and 10-h darkness/18°C. The material of ‘Bainong207’ which exhibited resistance during the adult stage, especially after the 4-leaf stage was inoculated with *Bgt* at the two-leaf stages. Leaves of five seedlings were collected at 0, 2, 6, 12, 24, 48, and 72 h after the inoculation. Total RNA was extracted using TRIzol reagent (Vazyme, China) in accordance with the manufacturer’s protocol.

### 2.2 Quantitative RT-PCR analysis of *TaPsbP* expression

The first-strand cDNA was synthesized with a HiScript^®^ III RT SuperMix for qPCR kit (Vazyme). The relative expression levels of the target genes in Bainong207 and Bainong AK58 were analyzed by qRT-PCR using an AceQ qPCR SYBR Green Master Mix (Vazyme) on the LC 480II system (Roche, Germany), and the wheat gene *TaTubulin* was used as an internal control. The program was executed as follows: 5 min at 95°C, then 40 cycles for 10 s at 95°C and 20 s at 60°C. The comparative 2^–ΔΔCT^ method was used to calculate the relative gene expression. The primers (**Supplementary Table S1**) were synthesized by Gene Create (Wuhan, China), and the raw qRT-PCR data are list in **Supplementary Table S2**.

### 2.3 BSMV-VIGS

To silence the corresponding *TaPsbP* genes, fragments of *TaPsbP2A-1* (*TaPsbP2B-1* and *TaPsbP2D-1*), *TaPsbP3A-1* (*TaPsbP3B-1* and *TaPsbP3D-1*), and *TaPsbP4A-1* (*TaPsbP4B-2* and *TaPsbP4D-2*) of 246 bp, 246 bp, and 237 bp, respectively, were amplifiedand then independently inserted into the γ-strain of *BSMV* to produce the recombinant vectors BSMV: *TaPSBP2A-1*, BSMV: *TaPSBP3A-1*, and BSMV: *TaPSBP4A-1*, respectively. The Chines elite wheat cultivar Bainong AK58, which is susceptible to the mixed *Bgt* in both seeding and adult stages was chosen as the receptor material in the BSMV-VIGS experiment to verify the function of the target genes. The second fully expanded leaves of ‘Bainong AK58’ were infected with the *in vitro* transcribed virus. BSMV: γ and BSMV: *TaPDS*- infected leaves served as controls. The infected plants were grown at 23°C, under a 14-h light/10-h dark cycle at 70% relative humidity. Leaves of the same age and position consistent with the controls showing symptoms of viral infection were cut and infected by *Bgt* for the disease resistance evaluation. The leaves used for powdery mildew disease evaluation were placed on a 6BA-plate with mixed *Bgt* spores and then cultured under conditions of 14-h light/22°C and 10-h/darkness 18°C for 6 days. qRT-PCR was used to evaluate the silencing efficiencies of the target genes. For the BSMV-VIGS assay, ‘Bainong AK58’ was grown at 16°C/12°C, under a 14-h light/10-h dark cycle at 70% relative humidity.

### 2.4 Identification of *PsbP* genes

All the Genome-wide data (Fasta and gff3 files) for *Triticum aestivum* (‘Chinese Spring’) were downloaded from IWGSC (http://www.wheatgenome.org/). Data for *Triticum urartu* (Tu 2.0) were downloaded from MBKBase (http://www.mbkbase.org/Tu/) (Ling et al., 2018). The data for *Hordeum vulgare* (IBSC_v2), *Triticum dicoccoides* (WEWSeq_v.1.0), *Aegilops tauschii* (Aet_v4.0), *Oryza sativa* Japonica Group (IRGSP-1.0), *Solanum lycopersicum* (SL3.0), *Arabidopsis thaliana* (TAIR10), *Vitis vinifera* (12X), and *Cucumis sativus* (ASM407v2) were downloaded from the Ensemble Plants (http://plants.ensembl.org/index.html). The typical PsbP domain (PF01789) was downloaded from the Pfam database (El-Gebali et al., 2019). Proteins were predicted using SMART (Letunic et al., 2021) and Conserved Domains (Lu et al., 2020), and then, the proteins containing intact PsbP conserved domains were retained (Hu et al., 2022a). All the gene names are listed in **Supplementary Table S3**.

### 2.5 Phylogenetic, gene duplication, gene structure, and conserved motif analysis

The phylogenetic tree was constructed using MEGA X with the Maximum-likelihood method (Kumar et al., 2018) and visualized by EvolView (He et al., 2016). Gene duplications were identified using MCScanX (Wang et al., 2012). The conserved motifs analysis was performed using the MEME program (http://memesuite.org/tools/meme). The parameters were as follows: the maximum number of motifs was set to 20 and the optimum width was 6–50 residues (Hu et al., 2022b). TBtools was used to visualize the gene structure (Chen et al., 2020). Syntenic relationships of gene pairs, gene duplication events, and the chromosome localization were determined by shinyCircos (Yu et al., 2018).

### 2.6 RNA-seq expression analysis

The RNA-seq data of 30 wheat *TaPsbP* genes after exposure to abiotic and biotic stresses were downloaded from WheatOmics10 (Ma et al., 2021). Expression pattern of the *TaPsbP* gene was visualized using TBtools (Chen et al., 2020).

### 2.7 PsbP protein structure and active sites

SPOMA was used to perform the secondary structure analysis of proteins (https://prabi.ibcp.fr/htm/site/web/app.php/home); SWISS-MODEL (https://swissmodel.expasy.org/) was used to perform the protein three-level structural prediction; SPPIDER (http://sppider.cchmc.org/) was used to predicted protein active sites (**Supplementary Table S4**); and the protein structure was visualized using PyMOL (Mooers, 2016).

## 3 Results

### 3.1 *PsbP* gene family members and classification

To more accurately determine the evolutionary relationships among the *PsbP* genes, six monocotyledonous plants, *O. sativa*, *T. aestivum*, *T. urartu*, *T. dicoccoides*, *Ae. tauschii* and *H. vulgare*, and four dicotyledonous plants, *A. thaliana*, *S. lycopersicum*, *V. vinifera*, and *Cucumis sativus*, were used. In total, 122 *PsbP* genes were identified in the six monocotyledons and four dicotyledons (**Tables 1**, **2**). Among the monocotyledons, nine *PsbP* genes were identified in rice. Thirty *PsbP* genes were identified in wheat, which were evenly distributed on the A, B, and D sub-genomes among chromosomes 2, 3, 4, 5, and 7. In total, 18 *PsbP* genes were identified in *T. dicoccoides*, with 9 in A and 9 in B sub-genomes. The *PsbP*s from the A sub-genome were distributed on chromosomes 2, 3, 4, and 7, and the *PsbP*s from the B sub-genome were distributed on chromosomes 2, 3, 4, 5, and 7 (**Table 1**). In the three diploid Triticeae species *T. urartu*, *Ae. tauschii*, and *H. vulgare*, 7, 11, and 6 *PsbP* genes, respectively, were identified. The *PsbP* genes of wheat-related species were mainly distributed on chromosomes 2, 3, 4, and 7. In wheat, *PsbP* genes were distributed 1:1:1 in the A, B, and D sub-genomes and the corresponding chromosomes, and in *T. dicoccoides*, *PsbP* was distributed 1:1 in the A and B sub-genomes, except for chromosomes 3 and 5, the *PsbP*s of the corresponding chromosomes of the A and B sub-genomes were also distributed 1:1.

**Table 1:** Numbers of *PsbP*s from five Triticeae species in each of the chromosomes.

Chromosome	*T. aestivum*			*T. dicoccoides*		*T.urartu*	*Ae. tauschii*	*H. vulgare*	Total
	A	B	D	A	B	A	D	H	
Chr.1	0	0	0	0	0	0	0	0	0
Chr.2	3	3	3	3	3	2	3	3	23
Chr.3	2	2	2	2	1	2	2	0	13
Chr.4	2	2	2	2	2	1	2	1	14
Chr.5	1	1	1	0	1	0	2	1	7
Chr.6	0	0	0	0	0	0	0	0	0
Chr.7	2	2	2	2	2	1	2	1	14
Un	0	0	0	0	0	1	0	0	1
Total	10	10	10	9	9	7	11	6	72

**Table 2:** Numbers of *PsbP*s in the ten analyzed species genomes in total and each class.

Genome	Total Number	Subclass							
		Class a	Class b	Class c	Class d	Class e	Class f	Class g	Class h
H. vulgare (HH)	6	1	1	1	1	1	0	0	1
T. urartu (AA)	7	1	1	1	1	0	1	0	2
Ae. Tauschii (DD)	11	2	2	1	1	1	1	1	2
T. dicoccoides (AABB)	18	2	3	2	2	1	2	2	4
T. aestivum (AABBDD)	30	3	6	3	3	3	3	3	6
O.sativa	9	2	2	0	1	0	1	1	2
A. thaliana	11	2	2	1	1	1	1	1	2
V. vinifera	11	2	2	1	1	1	1	1	2
S. lycopersicum	9	1	2	1	1	1	1	0	2
C. sativus	10	1	2	1	1	1	1	1	2
Total	122	17	23	12	13	10	12	10	25

**Figure 1:**
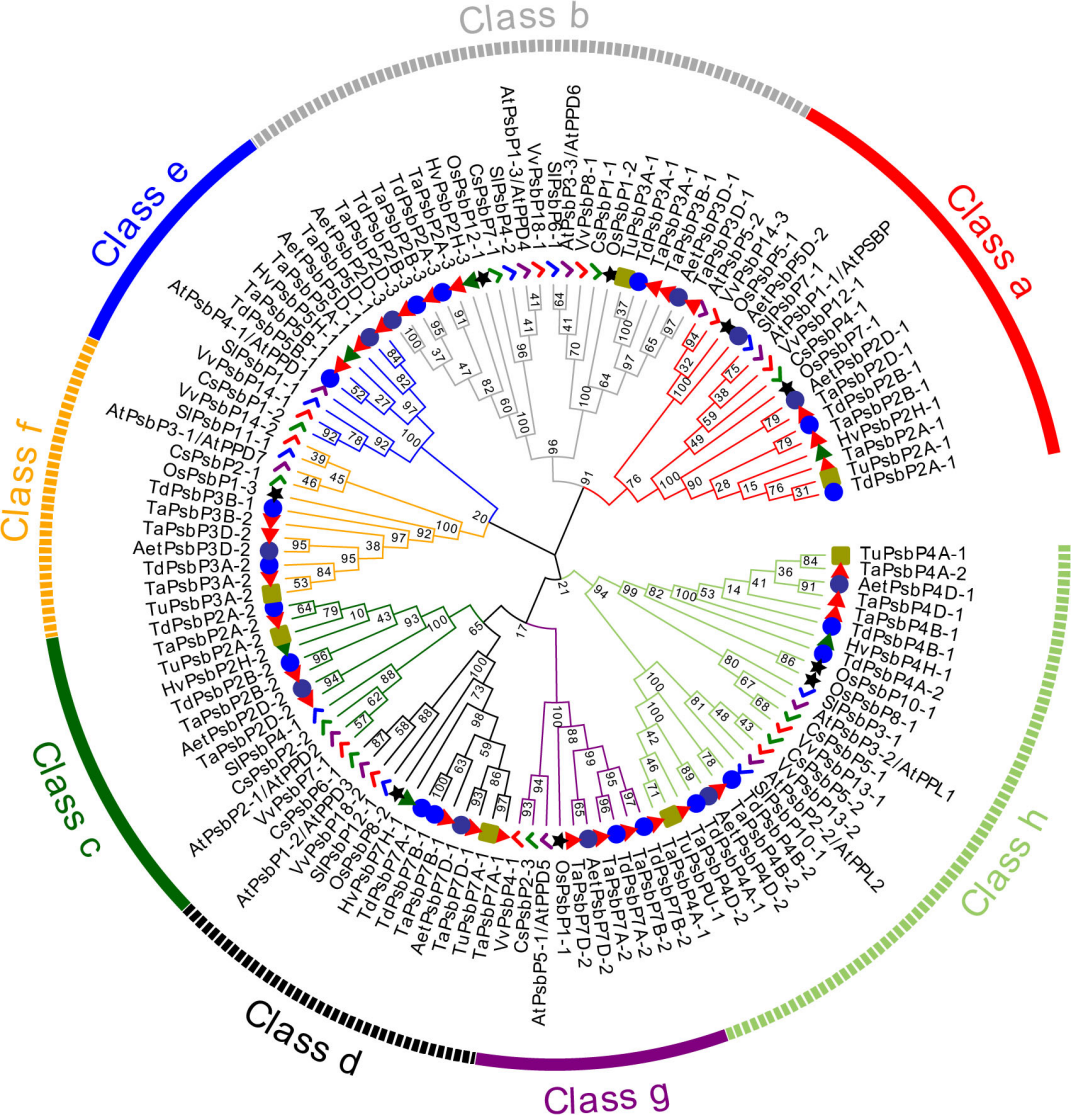
Phylogenetic analysis of PsbP protein in six monocotyledons and four dicotyledons. The PsbP proteins of different species were represented by different shapes and colors.

In dicotyledons, 11, 9, 10, and 11 *PsbP* genes were identified in *Arabidopsis*, *S. lycopersicum*, *C. sativus*, and *V. vinifera*, respectively. *Arabidopsis* previously appeared to contain 10 *PsbP* genes (Sato, 2010). However, here, 11 intact *PsbP* genes were obtained, and the new *PsbP* was named *AtPsbP5-2*. Using the *PsbP* gene classification system in *Arabidopsis* (Sato, 2010), the 122 *PsbP* genes were divided into 8 classes (Classes a–h), and *AtPsbP5-2* belonged to Class a (**Figure 1**). Each class contained *PsbP* genes of monocotyledons and dicotyledons, which indicates that the differentiation of *PsbP* genes may be earlier than that of divergence between monocotyledons and dicotyledons. Consequently, the *PsbP* genes of monocotyledons and dicotyledons in each class have undergone differential evolution. Among the monocotyledonous plants, the *PsbP* genes of *T. aestivum*, *T. dicoccoides*, and *Ae. tauschii* were distributed in all the classes. The *PsbP*s of *H. vulgare* was not found in Classes f and g, the *PsbP*s of *T. urartu* were not found in Classes e and g, and the *PsbP*s of *O. sativa* were not found in Classes c and Class e. Among the dicotyledonous plants, the *PsbP* genes of *Arabidopsis*, *V. vinifera*, and *C. sativus* were distributed in all the classes, whereas the *PsbP* genes of *S. lycopersicum* were not found in Class g (**Figure 1**; **Table 2**).

### 3.2 *PsbP* duplication and collinearity analysis

**Figure 2:**
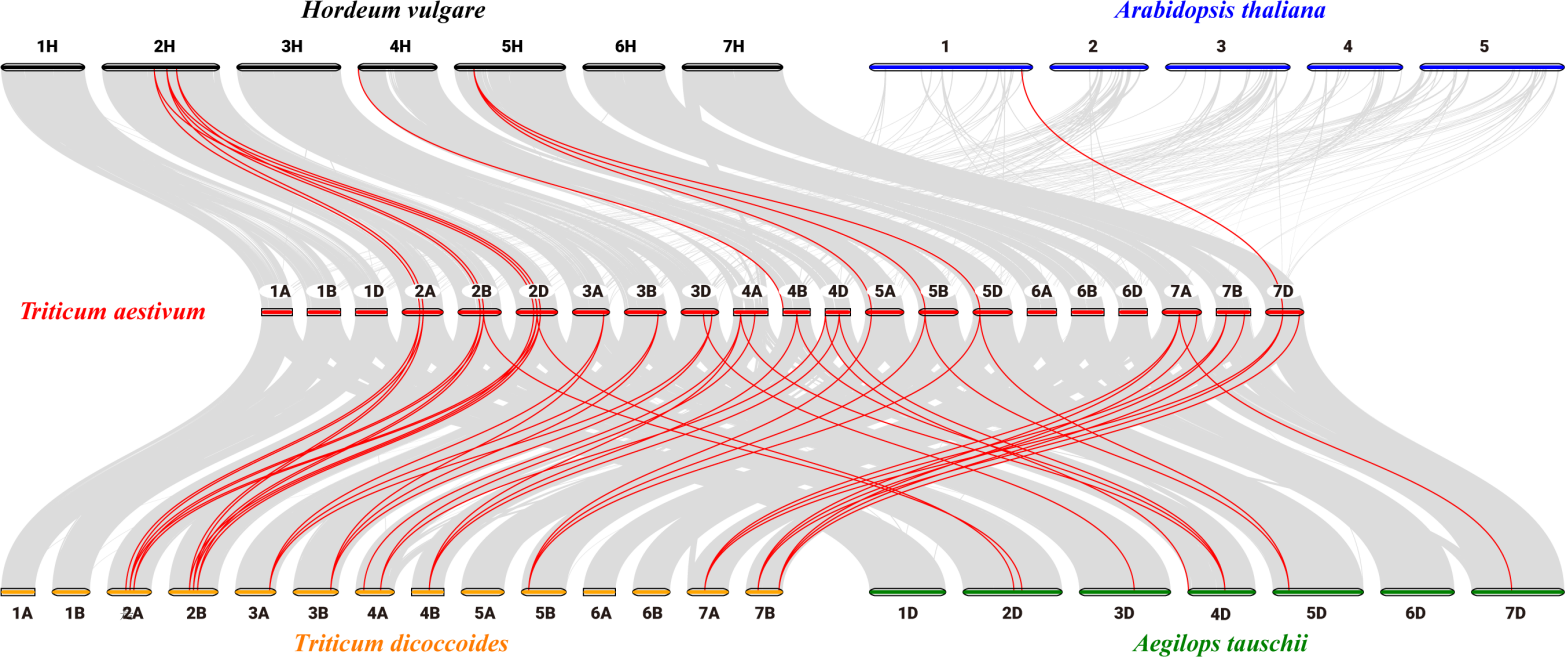
Collinearity analysis of *PsbP* genes between *Arabidopsis*, *H. vulgare*, *Ae. tauschii*, *T. dicoccoides* and *T. aestivum*.

**Figure 3:**
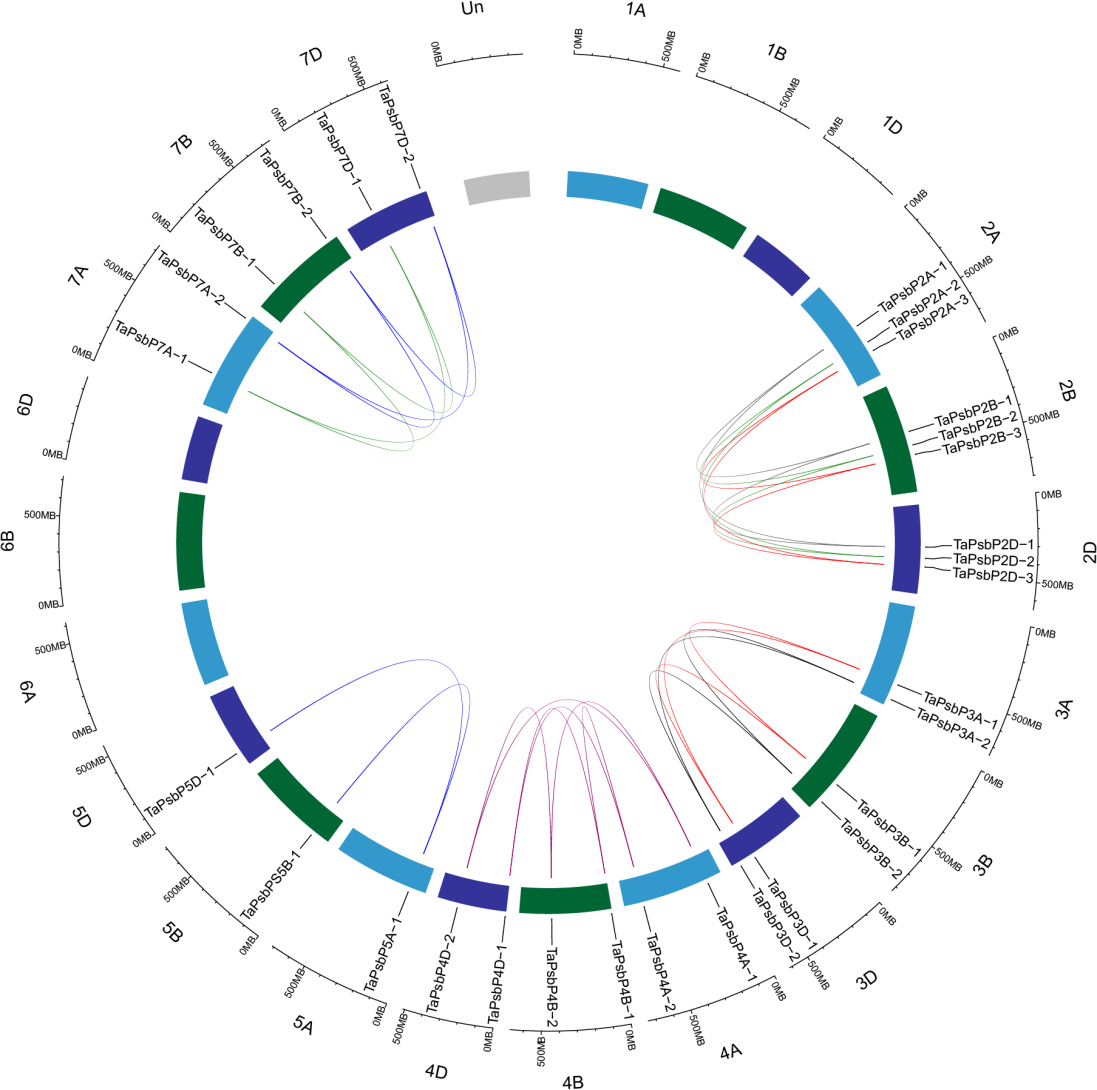
Location, homologous gene pairs and tandem duplication of *PsbP* genes in *T. aestivum*.

To clarify the relationships between *PsbP* genes in wheat and other species, and the differences in the *PsbP* genes between monocotyledons and dicotyledons, the collinearity of *TaPsbP* with *Ae. tauschii*, *H. vulgare*, *T. dicoccoides*, and *Arabidopsis* was studied (**Figure 2**). There were 37, 10, 11, and 1 *PsbP* gene pairs between wheat and *T. dicoccoides*, *Ae. tauschii*, *H. vulgare*, and *Arabidopsis*, respectively. Thus, the *PsbP* genes among the Triticeae species of wheat, *T. dicoccoides*, and *Ae. tauschii* have high similarity, whereas the *PsbP* genes between wheat and *Arabidopsis* have low similarity. Segment and tandem duplication analyses showed that there were no tandem duplications in wheat *PsbP*, whereas 29 pairs of segmental duplications were found of *TaPsbP*s (**Figure 3**). Therefore, *PsbPs* didn’t undergo gene expansion after the formation of hexaploidy common wheat.

### 3.3 PsbP conservative domains and gene structures

**Figure 4:**
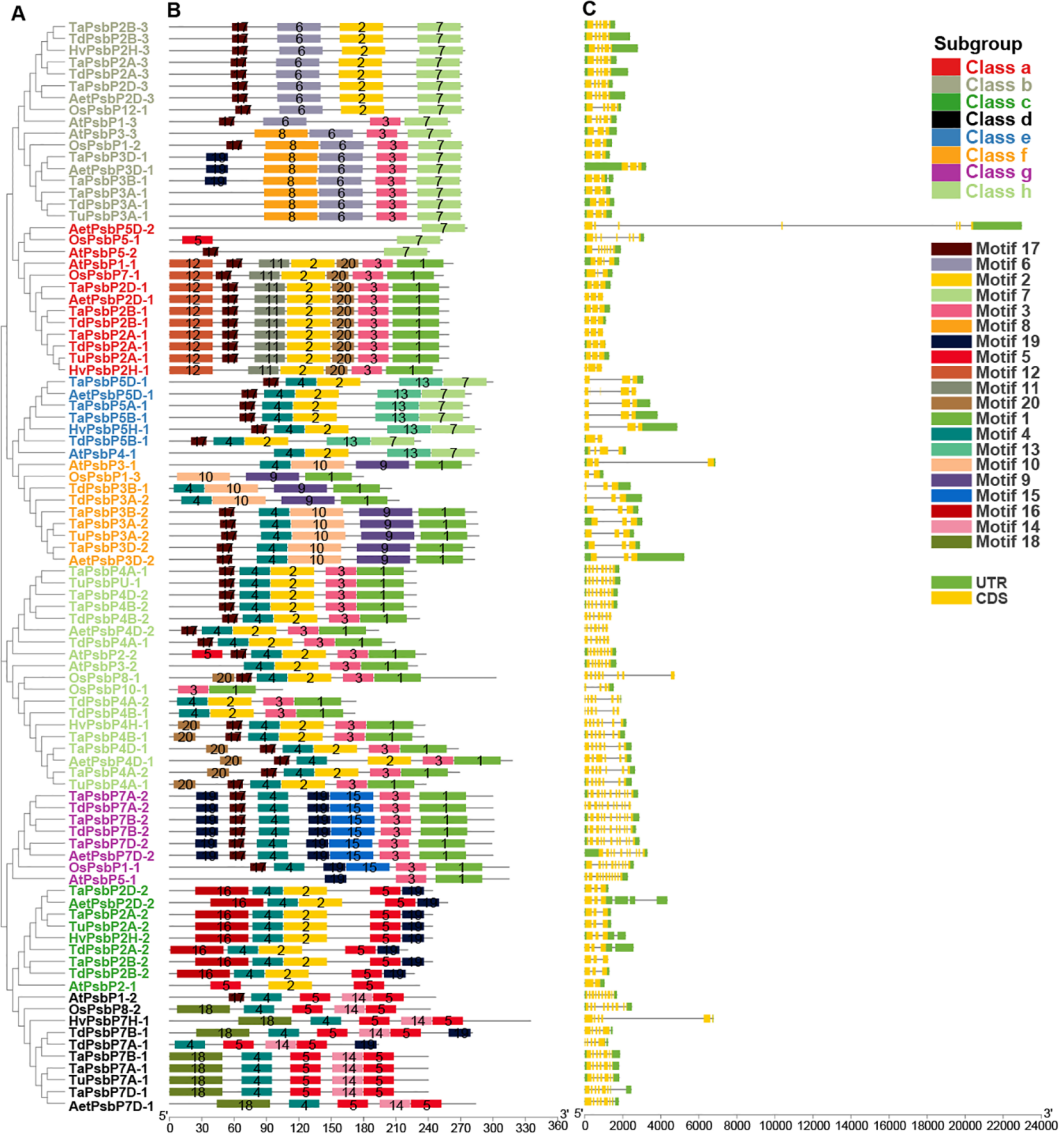
The gene structure and conserved domain organization of PsbP genes in monocotyledons and *Arabidopsis*. **(A)** The evolutionary tree was built by NJ method. **(B)** Motif distribution of PsbP proteins. **(C)** Exon–intron structure of *PsbP*s.

Intron/exon and conservative motif analyses of *PsbP* gene family members in different species are shown in **Figure 4**. The *PsbP* gene family members in the same species and the same class have similar motif compositions. In total, 10 PsbP proteins in Class a contained motifs 1, 2, 3, 11, 12, and 20, and the remaining three proteins all contained motif 7. Additionally, nine genes contained three exons, one gene contained four exons, and three genes contained seven exons (**Figure 4**). All the proteins in Class b contained motifs 6 and 7. Additionally, nine genes contained five exons and eight genes contained three exons. Monocotyledon PsbP proteins in Class c all contained motifs 2, 4, 5, 16, and 19. Additionally, all the genes contained three exons and two introns. *AtPsbP2-1* in Class c only contained motifs 2 and 5, and it contained two exons and one intron. In Class d, all the proteins contained motifs 4, 5, and 14. *AtPsbP1-2* contained 10 exons, which was the largest number in the analysis, and other members contained 5–9 exons. In Class e, the motifs 2, 4, 7, and 13 were all present, and members contained 2–5 exons. In Class f, all the PsbP proteins contained motifs 1, 4, 9, and 10, except *OsPsbP1-3*, which only contained motifs 1, 9, and 10. Additionally, eight genes contained three exons, and one gene contained two exons. In Class g, all the monocotyledon PsbP proteins contained motifs 1, 3, 4, 15, 17, and 19, whereas *AtPsbP5-1* in Class g only contained motifs 1, 3 and 19. The numbers of exons were either 10 or 11. In Class h, all the proteins contained motifs 1 and 3. Except for *OsPsbP10-1*, which contained 4 exons, the remaining *PsbP* genes contained 6–8 exons (**Figure 4**).

### 3.4 Expression pattern analysis of *TaPsbP*s

**Figure 5:**
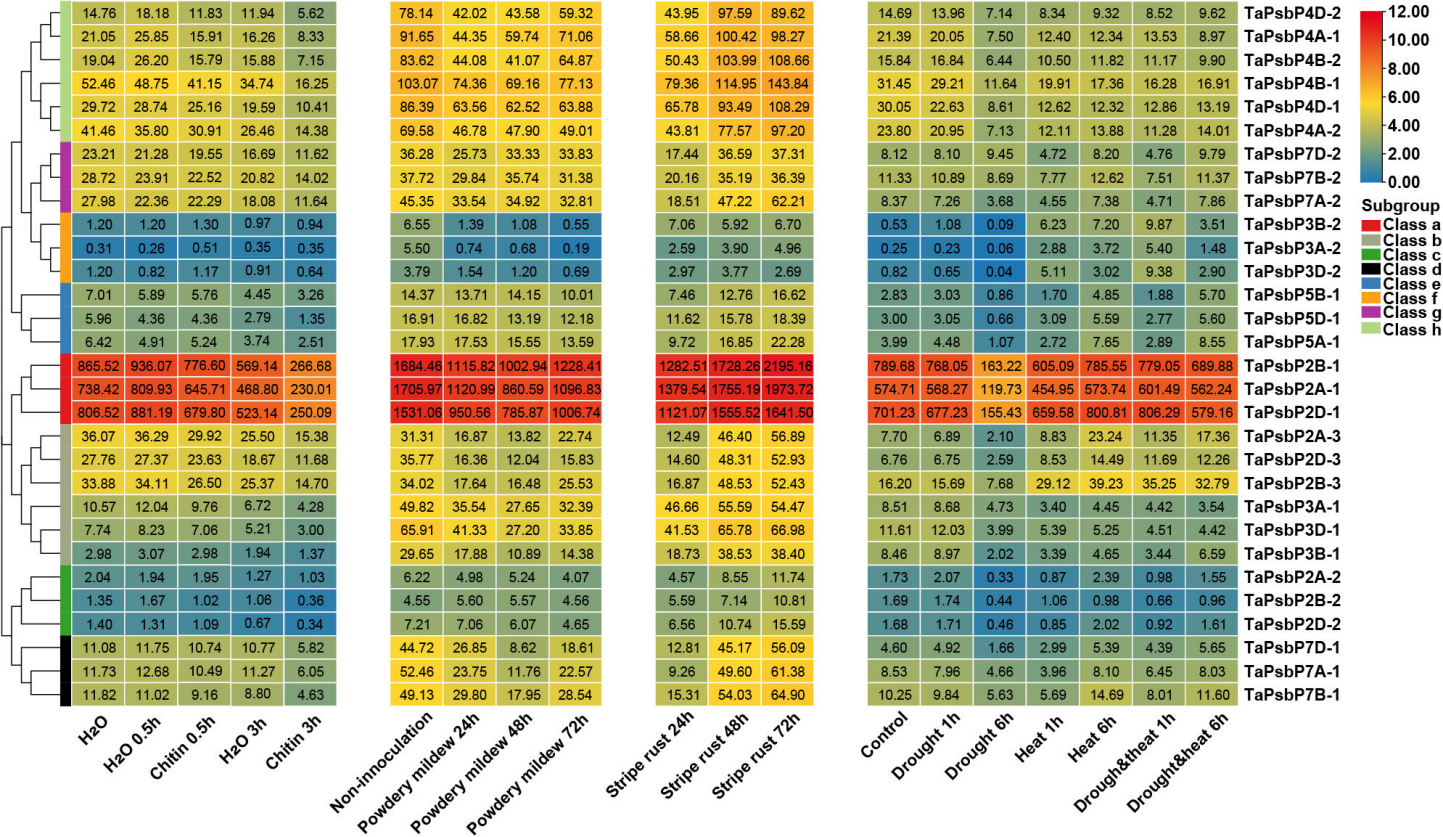
Heat map of the expression profiling of *TaPsbP* genes under different stresses. The color scale bar represents the expression values (in log2-based tags per million values) of the genes, and the values in square frames represent the tags per million values.

To elucidate the potential roles of *TaPsbP*s in different stresses, their expression patterns were studied using electron expression profiling. Wheat RNA sequence data were used to analyze the expression patterns under two abiotic stresses (drought and heat), under two biotic stresses (stripe rust and powdery mildew), and chitin treatments (**Figure 5**). After 6 h of drought stress, compared with the control, the expression levels of almost all the *TaPsbPs* genes were down-regulated. After 6 h of the heat stress, compared with the control, most of the gene in Classes a, c, d, and g were slightly changed. *TaPsbP2A-3*, *TaPsbP2B-3*, and *TaPsbP2D-3* in Class b and the *TaPsbP*s in Classes e and f were up-regulated, whereas *TaPsbP3A-1*, *TaPsbP3B-1*, and *TaPsbP3D-1* in Class b and the *TaPsbP*s in Class h were down-regulated. Under combined heat and drought stress, the expression patterns of almost all the *TaPsbP* genes were similar to those determined under just heat stress (**Figure 5**).

At 24 h after stripe rust infection most of the *TaPsbP* genes, except those in Class c were down-regulated compared with the control. In particular, the expression of *TaPsbP7A-1* was down-regulated to 17.6% and then returned to the pre-infection control level at 48 or 72 h (**Figure 5**). Under the stress of powdery mildew, compared with the control, the expression levels of all the *PsbP* genes showed down-regulated trends, with the levels of all the *PsbP* genes in Classes d and f and some of the *PsbP* genes in Classes a and b decreasing by more than 50% at 48 or 72 h. The expression level of *TaPsbP3A-2* decreased to 3.5% at 72 h. At 0.5 h after chitin treatment, the expression levels of most of the *TaPsbP* genes changed slightly, and some *PsbP* genes, such as *TaPsbP4A-1*, *TaPsbP4B-2*, *TaPsbP4D-2*, *TaPsbP3A-1*, *TaPsbP3B-1* and *TaPsbP3D-1* were down-regulated. At 3 h after treatment, the expression levels of most of the genes were down-regulated, such *TaPsbP4A-1*, *TaPsbP4B-2*, *TaPsbP4D-2* in Class h, and *TaPsbP2A-1*, *TaPsbP2B-1*, *TaPsbP2D-1* in Class a were down-regulated 50% compared with the control (water treatment at 3 h) (**Figure 5**). To more accurately study the functions of *PsbP* genes under powdery mildew stress, the *TaPsbP2A-1* (*TaPsbP2B-1* and *TaPsbP2D-1*), *TaPsbP3A-1* (*TaPsbP3B-1* and *TaPsbP3D-1*), *TaPsbP4A-1* (*TaPsbP4B-2* and *TaPsbP4D-2*), *TaPsbP4A-2* (*TaPsbP4B-1* and *TaPsbP4D-1*), and *TaPsbP7A-2* (*TaPsbP7B-2* and *TaPsbP7D-2*) genes, which respond to fungus or chitin induction, were selected for further qRT-PCR analyses.

**Figure 6:**
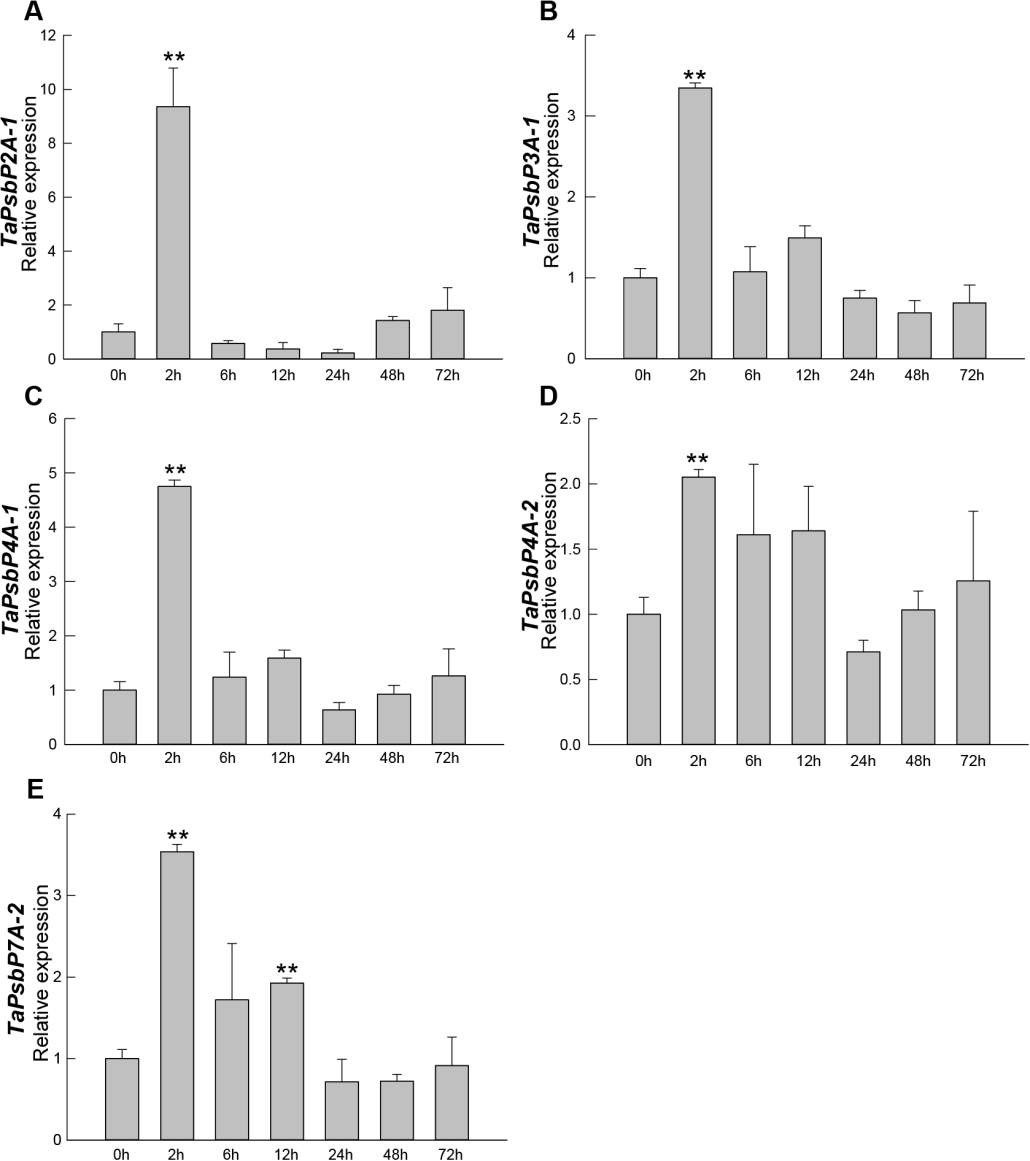
Expression analysis of *TaPsbPs* in Bainong207 under powdery mildew stress by qRT-PCR. **(A–E)** The response patterns of TaPsbP2A-1 **(A)**, TaPsbP3A-1 **(B)**, TaPsbP4A-1 **(C)**, TaPsbP4A-2 **(D)** and TaPsbP7A-2 **(E)** to Blumeria graminis f. sp. tritici (Bgt). These values are the average of three technical replicates in one biological replicate. Three biological replicates showed similar results. Duncan’s honestly significant difference test was used to analyze the significant differences. ** P <0.01. All the raw data for qRT-PCR are listed in **Supplementary Table S2**.

The relative expression patterns of five genes at different times during *Bgt* treatment are shown in **Figure 6**. The relative expression levels of *TaPsbP2A-1*, *TaPsbP3A-1*, *TaPsbP4A-1*, *TaPsbP4A-2*, and *TaPsbP7A-2* showed increasing and then decreasing trends. Compared with at 0 h, the relative expression levels of *TaPsbP2A-1*, *TaPsbP3A-1*, *TaPsbP4A-1*, *TaPsbP4A-2*, and *TaPsbP7A-2* increased significantly by 9.36, 3.25, 7.75, 2.05, and 3.54 times, respectively, by 2 h (**Figure 6**). Compared with at 0 h, the expression level of *TaPsbP7A-2* also significantly increased at 12 h, whereas the relative expression levels of the other four genes did not reach significant levels at other times.

### 3.5 *TaPsbP4A-1* negatively regulated powdery mildew resistance in common wheat ‘Bainong AK58’

**Figure 7:**
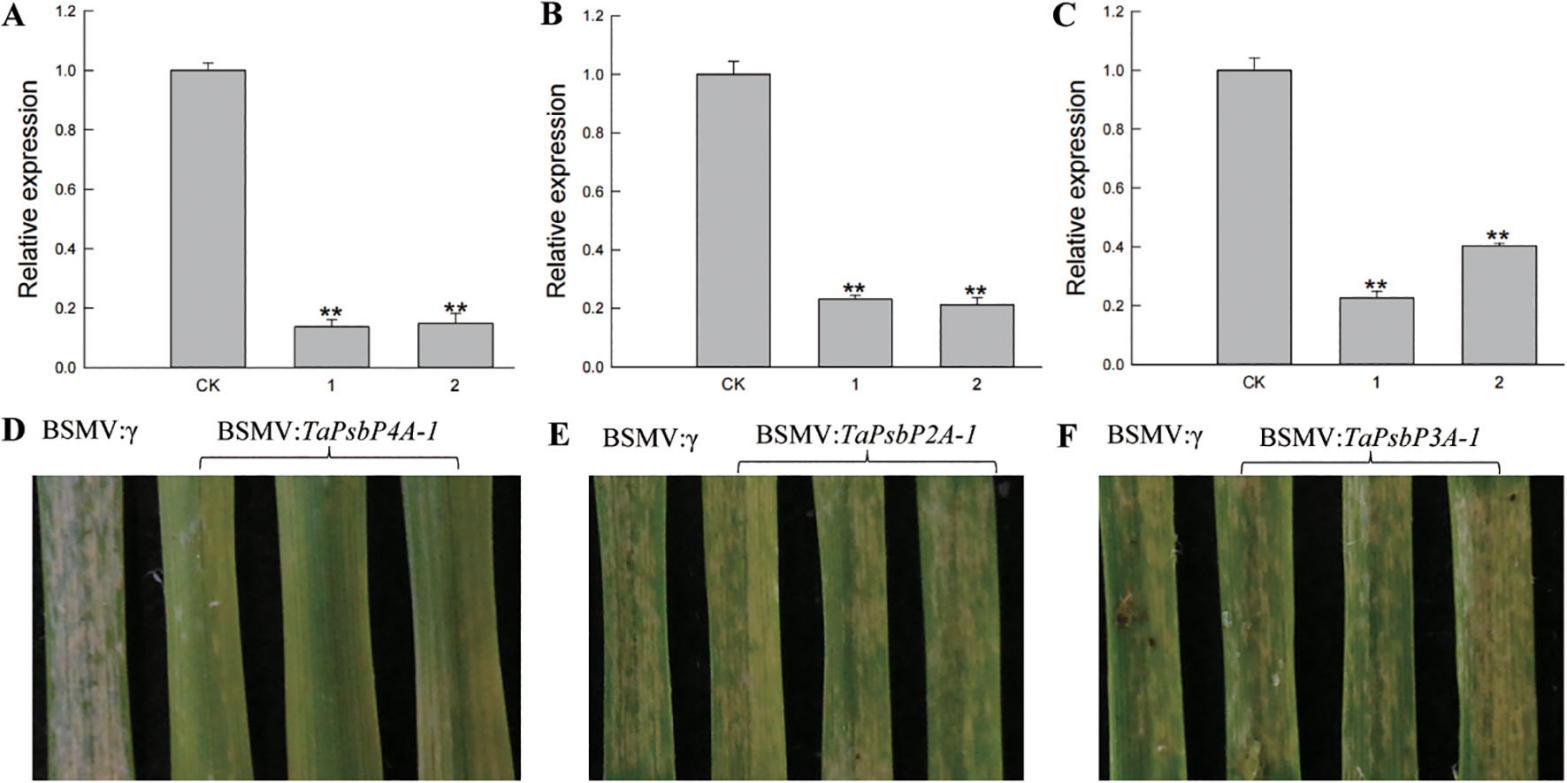
Functional analysis of *TaPsbP4A-1*, *TaPsbP2A-1* and *TaPsbP3A-1* by BSMV-VIGS in BainongAK58. Expression of *TaPsbP4A-1* **(A)**, *TaPsbP2A-1* **(B)** and *TaPsbP3A-1* **(C)** in the corresponding virus- infected leaves were checked by qRT-PCR. ** P < 0.01. BSMV: *TaPsbP4A-1* **(D)**, *TaPsbP2A-1* **(E)** and *TaPsbP3A-1* **(F)** infected plants were infected by *Bgt*, and photos were taken after six days of inoculation.

The relative expression levels of *TaPsbP2A-1* (*TaPsbP2B-1* and *TaPsbP2D-1*), *TaPsbP3A-1* (*TaPsbP3B-1* and *TaPsbP3D-1*) and *TaPsbP4A-1* (*TaPsbP4B-2* and *TaPsbP4D-2*) were induced by *Bgt* (**Figure 6**). *TaPsbP2A-1*, *TaPsbP3A-1*, and *TaPsbP4A-1* were selected to verify their potential roles in powdery mildew disease resistance in common wheat ‘Bainong AK58’. The qRT-PCR results showed that the expression levels of *TaPsbP4A-1*, *TaPsbP2A-1*, and *TaPsbP3A-1* in BSMV: *TaPsbP4A-1*, BSMV: *TaPsbP2A-1*, and BSMV: *TaPsbP3A-1*-infected plants, respectively, were significantly lower than those in the BSMV:γ-innoculated plants (**Figures 7A–C**). At 6 days after *Bgt* inoculation, BSMV: *TaPsbP4A-1-* infected leaves were more resistant to *Bgt* than those of BSMV: γ-infected plants (**Figure 7D**). There were no obvious differences in the responses to *Bgt* infection in *TaPsbP2A-1-* and *TaPsbP3A-1*- silenced leaves compared with those from controls (**Figures 7E, F**). Thus, *TaPsbP4A-1* may negatively regulate wheat powdery mildew resistance in ‘Bainong AK58’.

### 3.6 Analyses of PsbP protein structures and active sites

**Figure 8:**
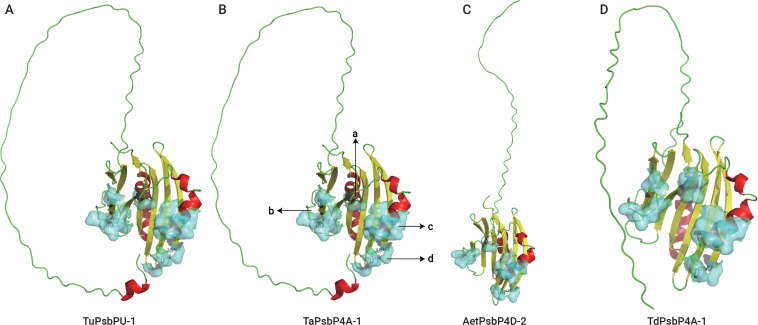
The protein structure and active sites of TuPsbPU-1 **(A)**, TaPsbP4A-1 **(B)**, AetPsbP4D-2 **(C)**, and TdPsbP4A-1 **(D)** from Triticeae species. Light blue coverage and amino acid labeling represent protein active sites, while the N-terminal irregularly curled active sites are not displayed. All active sites are listed in **Supplementary Table S4**. Active sites a and b being located at the junction of the beta turn, c being located at the alpha helix, and d being located at the junction of the alpha helix and beta turn.

**Figure 9:**
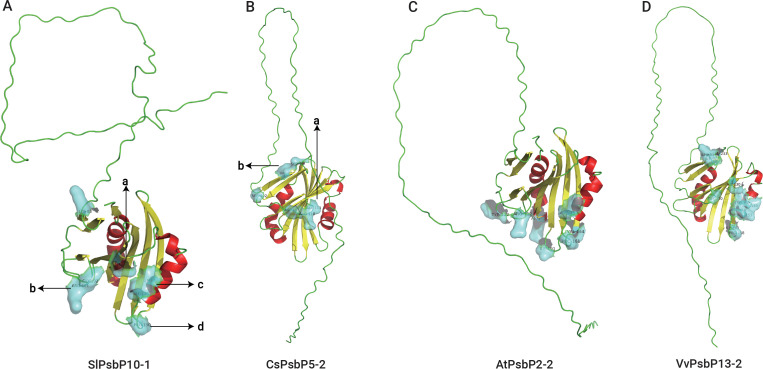
The protein structure and active sites of SlPsbP10-1 **(A)**, CsPsbP5-2 **(B)**, AtPsbP2-2 **(C)**, and VvPsbP13-2 **(D)** from dicotyledons. Light blue coverage and amino acid labeling represent protein active sites, while the N-terminal irregularly curled active sites are not displayed. All active sites are listed in **Supplementary Table S4**. Active sites a and b being located at the junction of the beta turn, c being located at the alpha helix, and d being located at the junction of the alpha helix and beta turn.

SWISS-MODEL was used to predict the tertiary structures and active sites of all the representative plant proteins on the same branch as TaPsbP4A-1 in the evolutionary tree (**Figures 8**, **9**, **Supplementary Table S4**). The protein tertiary structure and active site of TaPsbP3A-1 were also predicted (**Supplementary Figure S1**), but no active site was present for it. The tertiary structures of PsbPs in Class h were similar, irrespective of proteins being from monocotyledonous or dicotyledonous plants. TuPsbPU-1 and TaPsbP4A-1 contained four alpha helices, whereas the other analyzed proteins contained three alpha helices (**Figures 8**, **9**). Meanwhile, TaPsbP3A-1 contains 7 alpha helices (**Supplementary Figure S1**). The additional alpha helices of TuPsbPU-1 and TaPsbP4A-1 were located at the N-termini of the proteins (**Figures 8A, B**). In Triticeae species, AetPsbP4D-2 and TaPsbP3A-1 contained eight beta turns (**Figure 8C**, **Supplementary Figure S1**), whereas the other three proteins contained nine beta turns (**Figures 8A, B, D**). In dicotyledonous plants, SlPsbP10-1 contained nine beta turns (**Figure 9A**), whereas the other three proteins all contained eight beta turns (**Figures 9B–D**). This indicates that the homologous genes of monocotyledonous and dicotyledonous plants have similar structures but underwent slightly different evolutionary processes. In subgroup h, the active sites of PsbP in the Triticeae species are mainly concentrated in four areas (**Figure 8B**), with Active sites *a* and *b* being located at the junction of the beta turn, *c* being located at the alpha helix, and *d* being located at the junction of the alpha helix and beta turn. The four areas form a pocket-shaped activation site region. In dicotyledonous plants, SlPsbP10-1 also contained active sites in these four areas, but this was not universal (**Figure 9A**). The *a* and *b* active sites located at the beta turn junction are common, but the *c* and *d* active sites did not exist in CsPsbP5-2 (**Figure 9B**). There are generally less *c* active sites located in the alpha helices of other plants than in the Triticeae species (**Supplementary Table S4**). Although the protein structures are similar, the different active sites in PsbP proteins in different plants may lead to differences in the functions of homologous genes. Compared with TaPsbP4A-1 in subgroup h, the TaPsbP3A-1 protein in subgroup b had more structural alpha heles and no active sites, which may be the reason for they have similar expression pattern but show different functions under powdery mildew stress.

## 4 Discussion

Through a genome-wide analysis, 122 *PsbP* genes from 10 plant species were identified. The number of *PsbP* genes was directly correlated with the plant ploidy. Compared with a previous investigation (Sato, 2010), a new *PsbP* gene was identified in *Arabidopsis*, and named *AtPsbP5-2*. A phylogenetic analysis divided the *PsbP* genes into eight classes, with *AtPsbP5-2* belonging to Class a. Triticeae species *PsbP*s are distributed in each class, and the ratio of the gene numbers in *Ae. tauschii*, *H. vulgare*, *T. dicoccoides* and *T. aestivum* was determined to be 1:1:2:3. These proportions in species having different ploidy levels were similar to the chromosomal compositions. The *PsbP*s of *V. vinifera*, *C. sativus*, and *A. thaliana* were distributed among all the classes. However, there was no tomato *PsbP* gene in Class g, and the number of *PsbP* genes in tomato is one less than in the other three analyzed dicotyledonous plants.

Every class contained *PsbP* genes of monocotyledons and dicotyledons, which indicates that the differentiation of *PsbP* occurred earlier than that of divergence between monocotyledon and dicotyledon. Gene structures revealed that genes in the same class had similar motif combinations and similar numbers of introns and exons. Sequence and structural differences within the *PsbP* family reflect acquired functional variation (Sato, 2010; Jeh et al., 2023). *PsbP*s of common wheat were mainly acquired from ancestral donor species and by polyploidization, without tandem repeat expansion.

Powdery mildew is a serious fungal disease that affects wheat worldwide. Powdery mildew can occur at any stage of the wheat growth period, and after infection, it may cause leaf withering and yield losing (Xing et al., 2018; Hu et al., 2023). In the winter wheat region of China, the proportion of powdery mildew in the total sown area has increased annually owing to climate change (Tang et al., 2017; Hu et al., 2022a). Although some genes related to wheat powdery mildew have been identified, the disease’s pathogen mutates rapidly, resulting in many resistance genes becoming inefficacious. Mutation of the susceptibility genes or negative-controlled genes will confer broad-spectrum and durable resistance in plants, which is urgently-needed in plant (Van Schie and Takken, 2014). In recent years, with the development of gene editing, breeders and pathologist pay more attention to the new strategy to improve disease resistance by exploration and modification of the resistance negative-controlled genes (Oliva et al., 2019; Li et al., 2022).

Therefore, it is particularly important to explore regulatory genes related to wheat powdery mildew resistance and provide genetic resources for disease-resistance breeding. Some *TaPsbP* gene expression patterns from electron expression profile were different from qRT-RCR (**Figures 5**, **6**). This difference may be caused by the different wheat samples and pathogen races used in qRT-PCR and RNA-seq. *PsbP* plays an important role in plant immune processes. For example, the sensory plastid-related PsbP domain-containing protein 3 triggers plant growth and defense-related reactions (Jeh et al., 2023). Wheat Kinase START 1, which has a broad spectrum resistance to stripe rust, interacts with phosphorylates PsbO, an extrinsic member of PSII, to interfere with photosynthesis, then, it regulates leaf chlorosis and improves resistance to stripe rust (Wang et al., 2019). The accumulation of disease-specific protein during rice stripe virus infection leads to the recruitment of PsbP from chloroplasts into the cytoplasm, resulting in changes to the chloroplast structure and function. Silencing PsbP increases the severity of disease and viral accumulation (Kong et al., 2014). In addition to inhibiting positive immunomodulators, pathogens also control susceptibility factors that regulate plant immunity to promote colonization (Liu et al., 2021). The RXLR effector RXLR31154 targets and stabilizes host protein PsbP to control reactive oxygen species-mediated defense responses, thereby reducing plant defense responses and enhancing colonization (Liu et al., 2021). Previous study has shown that *Pm4a* allele on chromosome 2AL in Bainong AK58 showed race-specific resistance to *Bgt* (Xu et al., 2023). In this study, Bainong AK58 was susceptible to the mixed *Bgt* in both seeding and adult stages. The resistance of Bainong AK58 to mixed *Bgt* was significantly improved after the silencing of *TaPsbP4A-1*, indicating that *TaPsbP4A-1* negatively regulate wheat powdery mildew resistance. Further research is needed to investigate whether *Pm4a* affects the resistance conferred by *TaPsbP4A-1* silencing.

While the protein structures of homologous genes of the plants in the same branch as TaPsbP4A-1 were similar, the distributions of protein active sites in dicotyledonous plants and Triticeae species differed slightly. Structural differences are already common in duplicated genes and can produce homologous genes with different functions, but the mechanisms by which changes in protein structure promote functional differences remain unclear (Xu et al., 2012; Su et al., 2020). In particular, it is challenging to distinguish protein-disrupting variants from neutral variants (Brandes et al., 2023). Therefore, we speculate that for PsbP proteins in the TaPsbP4A-1 branch, differentiation in monocotyledonous and dicotyledonous plants produced functional differences owing to varied selection pressures, and these differences are reflected in the variations among the protein active sites, although they may have no significant impacts on the corresponding protein structures.

## 5 Conclusions

A total of 122 *PsbP* genes were identified from six monocotyledonous and four dicotyledonous species, and divided into 8 classes. The *PsbP*s in the same class have similar gene structures. No tandem repeat events were identified in wheat *PsbP* suggesting the *PsbP* genes in common hexaploid wheat were donated by diploid species. The wheat RNA-seq data showed that almost all the *PsbP*s were responsive to the induction by drought, heat, stripe rust, *Bgt* or chitin. The qRT-PCR showed that the expression levels of *TaPsbP2A-1*, *TaPsbP3A-1*, *TaPsbP4A-1*, *TaPsbP4A-2*, and *TaPsbP7A-2* were induced by *Bgt*. The silencing of *TaPsbP4A-1* increased the resistance of common wheat ‘Bainong AK58’ to *Bgt*, but the silencing of *TaPsbP2A-1* and *TaPsbP3A-1* have no obvious change in wheat powdery mildew resistance. This study provides valuable information for functional and evolutionary research on the *PsbP* gene family.

## Data Availability

The datasets presented in this study can be found in online repositories. The names of the repository/repositories and accession number(s) can be found in the article/**Supplementary Material**.

## Supplementary material

Supplementary Figure 1 The protein structure and active sites of the analyzed TaPsbP3A-1.

Supplementary Table 2 Raw data for qRT-PCR in Bainong207 and Bainong AK58.
