# Supplementary figures and images for: A newly identified photosystem II Subunit P gene TaPsbP4A-1 in Triticeae species negatively regulates wheat powdery mildew resistance
Source: Front Plant Sci. 2024 Nov 8;15:1452281. doi: 10.3389/fpls.2024.1452281 (PMC11581894; doi:10.3389/fpls.2024.1452281)

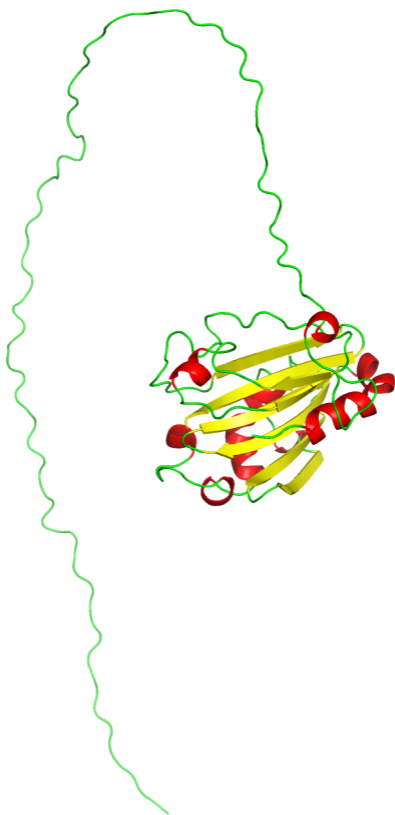

**TaPsbP3A-1**

Supplement: Supplementary Figure 1 — The protein structure and active sites of the analyzed TaPsbP3A-1. [file Image1.pdf]
