# Supplementary material for: A newly identified photosystem II Subunit P gene TaPsbP4A-1 in Triticeae species negatively regulates wheat powdery mildew resistance
Source: Front Plant Sci. 2024 Nov 8;15:1452281. doi: 10.3389/fpls.2024.1452281 (PMC11581894; doi:10.3389/fpls.2024.1452281)
Supplement: Supplementary Table 2 — Raw data for qRT-PCR in Bainong207 and Bainong AK58. [file Table1.docx]

**Table S1. The Primer sequences list.**

| Name | Sequence (5’-3’) | Used for |
| --- | --- | --- |
| *TaPsbP2A-1* -VIGS-F | TAGCTGAGCGGCCGCCCCGGGCGTCTGACTCGAACCCAC | Construct BSMV: *TaPSBP2A-1* vector |
| *TaPsbP2A-1*-VIGS-R | TAGCTGATTAATTAACCCGGGGGTTCAAACTGATGATCCC |  |
| *TaPsbP3A-1* -VIGS-F | TAGCTGAGCGGCCGCCCCGGGCACATAGCTGTAGTAAG | Construct BSMV: *TaPSBP3A-1* vector |
| *TaPsbP3A-1* -VIGS-R | TAGCTGATTAATTAACCCGGGCCCAAGTGCGCGGAGCCAT |  |
| *TaPsbP4A-1* -VIGS-F | TAGCTGAGCGGCCGCCCCGGGCTCGTTAGCCGTCACGA | Construct BSMV: *TaPSBP4A-1* vector |
| *TaPsbP4A-1* -VIGS-R | TAGCTGATTAATTAACCCGGGACAGACATTCGTGACCTA |  |
| *TaPsbP2A-1*-QF | CCAGCTTTTCGTCTGCAAG | qRT-PCR and gene silencing efficiency analysis |
| *TaPsbP2A-1*-QR | TGGCTGCTTCTCCGTAGG |  |
| *TaPsbP3A-1*-QF | GTGTTCTTCAAGGCCACCAC |  |
| *TaPsbP3A-1*-QR | GTCCTCGAATTTCACCTCCA |  |
| *TaPsbP4A-1-*QF | CAGCGTTCAAGGACCGTAAT |  |
| *TaPsbP4A-1-*QR | TCCAGTAGTTCCTGCCATCC |  |
| *TaTubulin*-F | ATCTCCAACTCCACCAGTGTCG |  |
| *TaTubulin*-R | TCATCGCCCTCATCACCGTC |  |
| *TaPsbP4A-2-*QF | CATGCACTTGGCACGATTAC | qRT-PCR |
| *TaPsbP4A-2* -QR | TGCATCAAATACCCCTCACA |  |
| *TaPsbP7A-2*-QF | GCTGATTGCGTTTTGTCTGA |  |
| *TaPsbP7A-2*-QR | GCCGATTTTGTTGGTGATTT |  |
